# Personalized Therapeutic Advances in Erythropoietin Signaling: From Anemia Management to Extensive Clinical Applications

**DOI:** 10.3390/pharmaceutics17091190

**Published:** 2025-09-12

**Authors:** Elena-Christen Creangă, Raluca Stan, Alina-Crenguţa Nicolae, Cristina Manuela Drăgoi, Ion-Bogdan Dumitrescu

**Affiliations:** 1 Faculty of Chemical Engineering and Biotechnology, National University of Science and Technology Politehnica Bucharest, Str. Gheorghe Polizu, nr. 1–7, Sector 1, 011061 Bucharest, Romania; elena.creanga@upb.ro (E.-C.C.); raluca.stan@upb.ro (R.S.); 2 Department of Biochemistry, Faculty of Pharmacy, “Carol Davila” University of Medicine and Pharmacy, 6 Traian Vuia St., 020956 Bucharest, Romania; alina.nicolae@umfcd.ro; 3 Department of Physics and Informatics, Faculty of Pharmacy, “Carol Davila” University of Medicine and Pharmacy, 6 Traian Vuia St., 020956 Bucharest, Romania; ion.dumitrescu@umfcd.ro

**Keywords:** EPO, rHuEPO, hypoxia-inducible factors (HIF), erythropoiesis-stimulating agents (ESA), gene doping

## Abstract

Erythropoietin (EPO) is a glycoprotein hormone essential for red blood cell production and a cornerstone therapy for anemia, particularly in chronic kidney disease. Beyond hematopoiesis, EPO exerts pleiotropic effects on metabolism, neuroprotection, and tissue regeneration. This review summarizes current insights into the molecular mechanisms, pharmacokinetics, and clinical applications of recombinant human EPO (rHuEPO) and its analogs, with emphasis on personalized therapeutic strategies. Emerging evidence highlights both therapeutic opportunities and risks, including resistance, cardiovascular complications, and misuse in sports doping. Advances in detection methods, pharmacogenomics, and the development of novel agents such as HIF-prolyl hydroxylase inhibitors are discussed, underscoring the expanding role of EPO in precision medicine.

## 1. Introduction

Erythropoiesis, the formation of red blood cells, is tightly regulated by erythropoietin (EPO), a hormone secreted mainly by renal interstitial fibroblast-like cells in adults and hepatocytes during fetal development. It governs the production of erythrocytes, with an estimated daily output of approximately 200 billion new red blood cells per individual [1]. The hormone binds with high affinity to erythropoietin receptors (EPORs) expressed on erythroid progenitor cells, thereby ensuring their survival, proliferation, differentiation, and maturation into fully functional erythrocytes [2].

EPO synthesis is predominantly stimulated by hypoxic conditions and occurs primarily in the interstitial fibroblasts of the adult kidney, whereas during fetal and perinatal development, it is produced mainly by perisinusoidal cells in the liver [1]. During early fetal development, neuroepithelial and neural crest cells serve as the main source of EPO, supporting primitive erythropoiesis [3]. EPO is classified as an erythropoiesis-stimulating agent (ESA), and its biological actions extend beyond hematopoiesis to include tissue protection in ischemic injury, regulation of metabolic processes, and modulation of physiological homeostasis [4].

The pharmacological attributes of ESAs have been exploited illicitly in sports to enhance performance by increasing the oxygen transport capacity of the blood. Advances in analytical techniques have facilitated the detection of EPO and its metabolites. Additional confirmatory assays have been developed, and anti-doping protocols continue to evolve accordingly [5].

Challenges in anti-doping efforts include the need for robust validation of detection techniques and the ongoing development of novel erythropoiesis-enhancing strategies, such as gene doping and the use of EPO analogs or mimetics [6]. Progress in anti-doping science has led to the refinement of detection technologies, including the replication and interpretation of metabolic signatures, remote testing methodologies, and the discrimination between endogenous compounds and structurally identical synthetic derivatives [7].

Clinical investigations on ESA dosing and the EPO resistance index are essential for understanding treatment efficiency and patients’ outcomes. They can guide adjustments to optimize safety and effectiveness of the treatment [8]. Future investigations should focus on assessing ESA-based therapeutic regimens, including their clinical effectiveness, safety profiles, and long-term outcomes related to placebo or untreated groups. These types of studies are essential for consolidating current evidence, optimizing treatment protocols, and guiding the development of future generations of ESAs with improved efficacy and safety [1].

## 2. Erythropoiesis-Stimulating Agents

EPO, a glycoprotein hormone composed of amino acids, is primarily synthesized in renal interstitial fibroblast-like cells in adults and to a lesser extent in the liver during the fetal development. In conditions of reduced tissue oxygenation (under hypoxic conditions) the synthesis takes place in other organs such as the spleen and bone marrow [1]. In individuals with advanced chronic kidney disease (CKD), especially those on dialysis, there is a marked decline in both EPO production and renal mass. To address this deficiency, treatment typically involves administering recombinant human erythropoietin (rHuEPO) alongside iron supplementation [8]. Epoetin alfa, a widely used ESA, is available under several brand names such as Eprex^®^, Epogen^®^, and Procrit^®^. Due to its relatively short biological activity—approximately 8 h—its administration must follow a carefully timed schedule based on its half-life to maintain consistent hemoglobin concentrations [9].

The principal stages involved in the production of ESAs are outlined in Table 1 [10]. Tradenames can vary by country and continent due to regional marketing strategies, regulatory approvals and historical licensing agreements.

**Figure 1:**
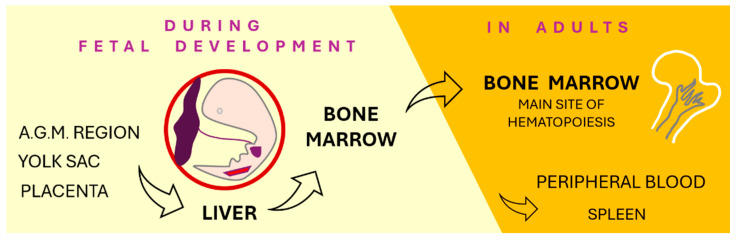
Schematic depiction of the human hematopoietic stem cell maturation pathway [16,17,18]. During fetal development blood stem cells appear in the Aorta-Gonad-Mesonephros (AGM) region, the yolk sac and placenta. These are the early sites where hematopoiesis begins. Later in the development process, the fetal liver becomes the main site where red blood cells form. Towards end of gestation, the bone marrow takes over the primary site for red blood cell formation. In adults, the bone marrow is the main location where hematopoietic stem cells produce all types of blood cells. The mature red blood cells enter the peripheral blood to circulate throughout the body. The spleen also contributes by filtering blood and supporting blood cell development.

ESAs are employed in a variety of therapeutic settings to promote red blood cell production, sustain adequate hemoglobin levels, and prevent erythropoietic resistance [11]. However, ESA administration is associated with certain risks, particularly in patients with underlying conditions such as hypothyroidism, vitamin B12 or iron deficiency, and disorders induced by chronic aluminum exposure (e.g., encephalopathy) [12]. Hematopoiesis, the process of blood cell formation, occurs in the bone marrow and encompasses leukopoiesis, erythropoiesis, and thrombocytopoiesis. Under physiological conditions, mature blood elements originate from hematopoietic stem cells via a tightly regulated sequence of proliferation and differentiation. These stem cells possess the ability to self-renew and differentiate into lineage-committed progenitors [13]. The EPO signaling cascade, activated under hypoxic conditions, stimulates erythroid progenitor cells, thereby promoting their survival, proliferation, differentiation, and hemoglobin synthesis [14]. Hematopoiesis is modulated within a highly organized bone marrow microenvironment—the stroma—through direct cell–cell interactions and the production of hematopoietic regulators such as erythropoietin and various cytokines. Additionally, this regulation involves less clearly defined components, such as megakaryocyte-derived growth factors, which play a crucial role in platelet formation through cytoplasmic fragmentation [15]. A schematic overview of the differentiation pathway of human hematopoietic stem cells is provided in Figure 1 [16].

A schematic representation of the developmental pathway of human hematopoietic stem cells (HSCs) illustrates their origin, migration, and differentiation potential. HSCs are primarily located in the bone marrow in adults and possess the ability to differentiate into various blood cell lineages. During embryogenesis, HSCs originate in the aorta-gonad-mesonephros (AGM) region—a critical site for definitive hematopoiesis. From there, they enter the circulatory system and migrate to extraembryonic tissues such as the yolk sac and placenta, where they continue their maturation. As development progresses, these cells acquire full hematopoietic potential and are capable of giving rise to all blood cell types. HSCs express specific surface markers, including GPI80 and PROM1 (also known as CD133), which help define their identity and function. Eventually, they colonize the bone marrow, which becomes the primary hematopoietic site. Throughout fetal development, HSCs are also present in the umbilical cord blood, as they traffic between the placenta and the fetal circulation, making cord blood a valuable source of stem cells at birth.

There is ongoing safety concerns associated with the use of ESAs, particularly regarding the role of ESA therapy in patients with reduced EPO production. To evaluate the safety profile of ESAs in dialysis patients, dosing strategies and resistance indices have been examined. Moreover, several pathological conditions resulting from either insufficient or excessive EPO production have been characterized. These findings have supported the development of innovative therapeutic agents that improve the safety of ESA use, particularly in the management of hematological malignancies [8].

With the expiration of patents for originator ESAs, biosimilar products have emerged as cost-effective alternatives. Biosimilars (EPO analogs) are designed to be highly similar to the original ESAs in terms of analytical structure, pharmacokinetic properties, and therapeutic efficacy. In 2006, the European Medicines Agency (EMA) introduced a comprehensive guideline to support the development and approval of biosimilar medicines, which continues to be regularly updated. According to this regulatory framework, an approved biosimilar must demonstrate high similarity to the reference biological product in all critical aspects.

The regulatory framework governing EPO analogs encompasses several critical aspects, including molecular structure, safety profile, immunogenicity, pharmacological, toxicological and pharmacokinetic evaluations, as well as biological assessments and post-marketing surveillance of the approved products [19]. The use of EPO derivatives has been expanding alongside systematic monitoring of adverse effects—such as cardiovascular events, myocardial infarction, stroke, and renal failure—through rigorous clinical pharmacovigilance studies. Dedicated research emphasizes the therapeutic potential of biosimilar ESAs, promoting broader access to treatment while simultaneously supporting the development of alternative therapies for patients who exhibit resistance to conventional ESA-based regimens [20]. Clinical trials and observational studies indicate that EPO biosimilars can achieve comparable hemoglobin (Hb) targets to the reference EPO products in patients with anemia linked to CKD or induced chemotherapy. The adverse effects found such as immunogenic reactions, thromboembolic events and antibody formation are similar to those of seen with innovator EPO. Post marketing surveillance confirms no meaningful differences in safety and efficacy. As a result, biosimilars can be considered reliable alternatives to anemia management [21].

## 3. The Hematopoietic Role of Erythropoietin in the Era of Personalized Medicine

EPO plays a crucial role in regulating erythropoiesis throughout fetal, neonatal, infant, and adult life—though not during the embryonic stage. As mentioned before, EPO production is upregulated by renal interstitial cells through a mechanism involving hypoxia-inducible factor-2α (HIF-2α). As a result, in adults approximately 90–95% of EPO is synthesized in the kidneys, with the liver contributing the remaining portion [22]. HIF also assists immune cells in adapting to the hypoxic environments typically found in infected or inflamed tissues [23].

Renal oxygen sensing relies on Hb concentration; when oxygen levels drop, such as during hypoxia, EPO production is accelerated. EPO binds to EPORs, thereby stimulating erythrocyte production and enhancing tissue oxygen delivery [24]. The coordination of EPO synthesis in the kidneys and liver, along with iron homeostasis, is governed by HIF and further regulated by HIF-prolyl hydroxylases (HIF-PHs) [25]. Beyond its hematopoietic role, EPO also contributes to glucose metabolism and the regulation of systemic energy balance [26].

Transcription factors, particularly HIFs, orchestrate the cellular response to low oxygen availability. Under hypoxic conditions, HIFs activate a transcriptional program that enables the organism to adapt both functionally and metabolically, including promoting vascular remodeling. HIF is a heterodimeric complex consisting of one of three oxygen-sensitive subunits—HIF-1α, HIF-2α, or HIF-3α—paired with the constitutively expressed HIF-1β. Among these, HIF-2α plays a pivotal role in regulating the erythropoietic response to hypoxia [27].

Under normoxic conditions, HIF-α subunits undergo proline hydroxylation, a modification catalyzed by the iron-dependent enzyme prolyl hydroxylase (PHD), which targets them for proteasomal degradation. In contrast, hypoxic conditions inhibit this degradation, allowing HIF-α to stabilize and accumulate. Specifically, HIF-2α becomes upregulated in erythropoietin-producing renal cells, where it directly regulates the transcription of the erythropoietin gene. Mutations in HIF-2α are associated with elevated red blood cell production, reflecting its role in enhancing EPO synthesis. On hematopoietic cells responsive to EPO—particularly early-stage erythroid progenitor cells—EPORs are highly expressed. This receptor density facilitates fine-tuned EPO signaling, promoting the survival, proliferation, and differentiation of erythroid progenitors into mature red blood cells.

EPO binds to EPORs on the surface of erythroid progenitor cells, initiating a cascade that includes tyrosine phosphorylation. Tyrosine, an amino acid, plays a crucial role in cellular function regulation and energy metabolism. Through this interaction, EPO modulates EPOR expression, with receptor levels being dynamically adjusted throughout the stages of erythroid differentiation [1]. Additionally, EPO binding to the homodimeric EPOR activates downstream signaling pathways essential for erythropoiesis. Beyond hematopoietic regulation, EPO also influences the hypothalamic-pituitary axis, contributing significantly to glucose metabolism and systemic energy balance [26].

Personalized medicine represents a paradigm shift from “one-size-fits-all” approaches to strategies tailored to individual variability in genes, environment, and lifestyle. Within this framework, erythropoietin (EPO) and its analogs have gained renewed interest not only in treating anemia but also in a variety of non-hematologic conditions, including neurodegeneration, metabolic dysfunctions, and ischemic injuries [28].

The efficacy of EPO therapy depends heavily on individual patient factors such as EPOR gene polymorphisms, inflammatory state, iron metabolism, and comorbidities. EPO exerts its biological effects via binding to EPORs, activating JAK2/STAT5, PI3K/Akt, and MAPK signaling pathway [29]. Emerging evidence suggests variability in EPOR expression and function based on genetic polymorphisms (e.g., rs1617640 in the EPO gene) that can influence therapeutic response [30]. Understanding these molecular variations can inform the optimal selection and dosing of ESAs or rHuEPO in individualized protocols.

EPO resistance is a significant challenge in patients with chronic kidney disease or inflammatory diseases. Pharmacogenomic profiling can identify genetic variants associated with hypo-responsiveness to EPO [31]. Factors such as hepcidin overexpression, inflammatory cytokines (e.g., IL-6, TNF-α), and HIF pathway dysregulation are critical in modulating EPO effectiveness [32]. Integration of omics data facilitates the identification of biomarkers that predict treatment response [33].

Recent advances have demonstrated EPO’s neuroprotective, cardioprotective, and metabolic regulatory effects. In the central nervous system, EPO modulates neuroinflammation, prevents apoptosis, and enhances neurogenesis [34]. In the cardiovascular system, it supports endothelial repair and NO synthesis [35]. These non-erythropoietic effects depend on tissue-specific EPOR isoforms and local hypoxic responses—offering unique opportunities for personalized therapeutic interventions in stroke, heart failure, and metabolic syndrome [36].

The advent of EPO analogs (e.g., epoetin zeta, darbepoetin alfa) offers cost-effective alternatives to originator EPOs. Comparative immunogenicity, pharmacokinetics, and clinical outcomes need to be carefully considered [37]. Personalized medicine enables the selection of the most appropriate EPO variant based on patient-specific risk factors, such as autoimmune tendencies or prior hypersensitivity to certain formulations.

New therapeutic avenues include HIF-PHIs and gene therapy approaches that modulate endogenous EPO production. Roxadustat, daprodustat, and vadadustat represent promising oral agents that restore EPO levels in a physiologically regulated manner [38]. Gene-editing strategies, such as CRISPR-Cas9-mediated modulation of HIF pathways, offer potential for long-term correction of anemia in select patient populations [39].

Personalized EPO therapy must balance efficacy with safety. Monitoring for thrombotic events, cardiovascular risk, and long-term effects on tumor progression is essential [40]. Ethical concerns arise in gene doping and off-label uses, particularly in performance enhancement. Stratifying patients using AI-guided risk models could enhance safety and precision [41].

## 4. Erythropoietin Beyond Hematopoiesis: Exploring Its Non-Hematopoietic Functions

### 4.1. Non-Hematopoietic Roles of EPO

EPO is a pleiotropic cytokine whose biological activity extends beyond the regulation of erythropoiesis. It binds to and activates EPORs not only on hematopoietic cells but also on various non-hematopoietic tissues. Current literature indicates that EPO is involved in numerous physiological processes, including angiogenesis, cardioprotection, neuroprotection, stress response modulation, anti-inflammatory actions, energy metabolism regulation, maintenance of homeostasis, and control of oxidative metabolism [42]. Since the introduction of rHuEPO in 1989, pharmacological research has increasingly recognized EPO’s functions in non-hematopoietic systems [43]. Additional roles have been attributed to EPO, such as acting as a biological mediator, a neurotransmitter involved in cell proliferation, and a regulator of vascular tone, blood pressure, and tissue and pulmonary oxygenation [44].

EPO is also expressed in endothelial cells, cardiomyocytes, and muscle cells, where it plays a protective role by preventing apoptosis and reducing collagen deposition in ischemic myocardial tissue [45]. The cardioprotective and vasculoprotective effects of EPO are reflected through its ability to promote neovascularization, vasodilation, and endothelial repair, partly by stimulating nitric oxide (NO) production [46]. EPO’s neuroprotective properties are evidenced by its capacity to improve cerebral lesions and enhance brain revascularization [47]. To enhance these effects, numerous EPO derivatives are being engineered to increase their affinity for EPORs, thereby augmenting their neuroprotective potential [48].

RHuEPO is also recognized for its neuroprotective and neurorestorative properties, particularly in the context of the premature brain. It has been shown to reduce white matter injury and decrease the need for red blood cell transfusions in preterm infants. Nevertheless, only a limited number of controlled studies have assessed its long-term impact on neurodevelopmental outcomes [49]. The pleiotropic effects of rHuEPO are well-documented across multiple tissues and organs, including the central nervous system. Due to its pharmacological properties, rHuEPO is emerging as a promising neuroprotective candidate for conditions such as Alzheimer’s disease (AD). Neuroscience researchers increasingly propose that rHuEPO and its molecular derivatives may offer a novel therapeutic approach for neurodegenerative disorders. Enhancing the endogenous EPO/EPOR system through exogenous EPO administration could provide crucial protection against neurological damage induced by pathological Aβ and tau protein aggregates or other neurotoxic factors implicated in AD pathogenesis [50].

**Figure 2:**
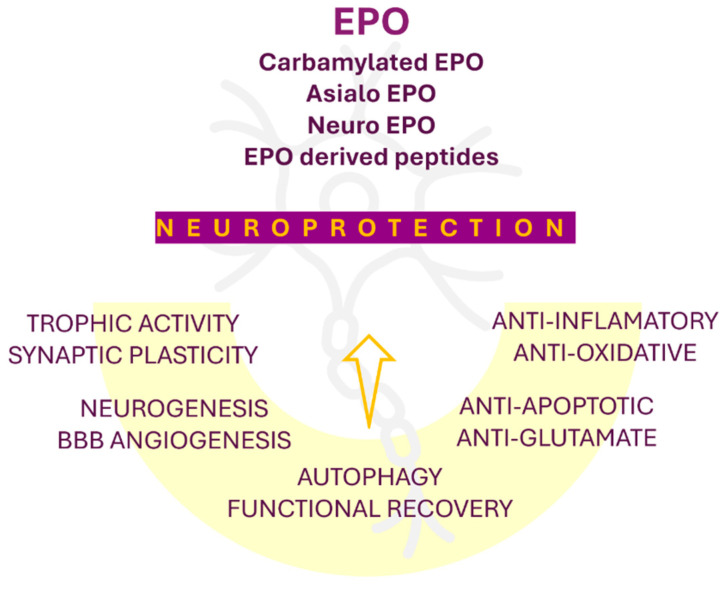
Graphic representation of the neuroprotective effect of EPO, EPO derivatives or alternative molecules [51,52,53]. Biological effects of EPO and its derivatives, highlighting their neuroprotective, regenerative roles including: promotion of synaptic plasticity, neurogenesis, angiogenesis, autophagy, functional recovery, as well as anti-inflammatory, anti-oxidative, anti-apoptotic and anti-glutamate actions.

However, excessive administration of EPO may result in elevated hematocrit levels, potentially leading to adverse effects such as hypertension and increased blood viscosity. The neuroprotective actions of EPO and its derivatives are illustrated in Figure 2. Clinical studies have also demonstrated that EPO derivatives may confer tissue-protective benefits by enhancing muscle protein synthesis [51].

EPO plays a significant role in muscle development and remodeling, contributing to an increased number of skeletal muscle fibers and enhancing physical performance capacity [54]. Additionally, EPO is involved in the regulation of lipid metabolism and glycemic control. It has been studied in the context of diet-induced obesity, a condition often linked to insulin resistance and impaired glucose tolerance—key indicators of potential diabetic risk [55,56]. In such settings, EPO supports metabolic homeostasis by eliciting anti-inflammatory effects, modulating glucose metabolism, and reducing fat accumulation [57].

EPO plays a critical role in regulating energy metabolism and the expression of EPORs in white adipose tissue (WAT). Studies have shown that male mice lacking EPORs specifically in adipose tissue exhibit increased fat mass and a heightened susceptibility to diet-induced obesity [58]. EPO/EPOR signaling is also crucial for maintaining the balance between osteogenesis and adipogenesis [59].

In the context of diet-related obesity, EPO administration exerts anti-inflammatory effects by inhibiting proinflammatory cytokines and reducing macrophage activity [60]. Furthermore, EPO therapy targeting adipose tissue inflammation has been associated with elevated hematocrit levels while maintaining stable body weight, and it contributes to improved insulin sensitivity and glucose tolerance, thereby promoting overall metabolic homeostasis [61].

Ruan et al. demonstrated in their study [62] that itaconate—a mitochondrial metabolite with anti-inflammatory properties—activates the antioxidant transcription factor Nrf2 (nuclear factor erythroid 2–related factor 2), thereby reducing inflammation and supporting the differentiation of murine stress erythroid progenitors (SEPs) [63].

This suggests that itaconate and EPO may act synergistically during stress-induced erythropoiesis [61]. In the skeletal system, EPORs play a functional role in the development of osteoblasts and osteoclasts, which are essential for bone remodeling, as well as in the differentiation of osteoblasts, adipocytes, and chondrocytes. The impact of EPO on bone remodeling is age-dependent, with reduced EPO production being more closely linked to diminished osteoblast formation than to changes in osteoclast activity [64]. Moreover, EPO contributes to the regulation of skeletal stem cell differentiation and development, even in the absence of endogenous EPO–EPOR signaling [65].

### 4.2. Clinical and Preclinical Validation of EPOs Non-Hematopoietic Effects

The non-hematopoietic effects of EPO can be validated preclinically, as well as clinically. Distinguishing between clinical and preclinical validation for non-hematopoietic EPO effects is crucial for ensuring therapeutic efficacy and patient safety. Preclinical studies involve cell cultures and animal models, being able to establish the foundational evidence for EPO’s broad therapeutic potential beyond red blood cell production. Clinical trials are conducted on humans and can confirm whether these effects translate to safe and efficient treatment in diverse patient populations (for specific conditions). This distinction is relevant because findings in animal models do not always predict human outcomes and are able to predict significant risks, such as adverse cardiovascular events.

The neuroprotective effects of EPO are preclinically validated in rats subjected to cardiac ischemia injury. The results of the study [66] suggested that EPO has anti-oxidative, anti-inflammatory and anti-apoptotic effects on the brain following cardiac injury. The neuroprotective properties of EPO are also clinically validated. Studies [67] investigated the potential benefits of EPO in patients suffering from acute ischemic stroke (a medical condition associated with high risk of mortality and disability to conventional therapies). As follows, EPO doses (of 5000 IU each) given 48 and 72 h after the stroke showed neuroprotective benefits and durable improvements [67]. The cardioprotective effects of EPO are demonstrated in recent preclinical studies in rat models of ischemia–reperfusion injury. EPO is able to activate the Akt/glycogen synthase kinase 3 beta (GSK-3β) pathway in order to ensure cardioprotection. As follows, an acute treatment with EPO improved functional cardiac recovery, reduced the infarct size and lowerd the creatine kinase release. It was also able to preserve the energy metabolism, including ATP production and glycogen stores, maintain mitochondrial integrity, and support cell survival. Clinical validation is limited [46]. Beyond the clinical use of EPO for treating anemia, EPO can influence glucose and fat metabolism in adipose tissues, skeletal muscle and the liver. It is able to enhance lipolysis and suppress fat storage through the EPO/EPOR-RUNX1 pathway. Furthermore, non-erythroid EPOR agonists like ARA290 are able to demonstrate in mouse models that these metabolic effects can occur independently of red blood cell production. Because of that, EPO is able to ensure metabolic homeostasis and to preserve whole-body energy. Clinical data on this behalf is limited [68].

### 4.3. Bridging EPO Functions and Gene Expression

While EPO is well known for its role in red blood cell production, recent research has highlighted its diverse effects on non-hematopoietic tissues, including: cardioprotection, metabolic regulation, neuroprotection and tissue repair [42,43,44,45,46,47,48,49,50,51]. These pleiotropic functions [50] can depend not only circulating levels of EPO but also on regulated gene expression in response to physiological and environmental cues (for, e.g., hypoxia). Understanding the mechanisms that control EPO gene expression is therefore essential for science and clinical applications. It can provide a foundation for exploring therapeutic and non-therapeutic manipulations, including rHuEPO in the context of gene doping.

## 5. Modulation of Erythropoietin Gene Expression

As mentioned before, EPO is a glycoprotein hormone essential for regulating erythropoiesis—the production of red blood cells. It is primarily produced by the kidneys (interstitial fibroblast-like cells), although during fetal development the liver is the major site of synthesis.

The expression of EPO is tightly controlled to ensure that red blood cell mass and oxygen transport match the body’s metabolic demands. Oxygen availability is the primary regulatory signal, although inflammatory, hormonal and epigenetic factors also influence EPO production. Furthermore, the key oxygen-sensing mechanism is the HIF pathway. Under normoxic conditions, HIF-α subunits are hydroxylated by PHDs, which allows the von Hippel-Lindau (VHL) complex to recognize them and mark them for breakdown by the cell [69].

Under hypoxic conditions, PHD activity is suppressed, stabilizing HIF-α, which translocates to the nucleus, dimerizes with HIF-β, and binds to hypoxia response elements (HREs) on the EPO gene promoter, enhancing transcription. Other modulators include inflammatory cytokines, androgens, and specific signaling molecules like PI3K and MAPK pathways [70,71]. As mentioned above, hydroxylation of HIF-α subunits, a post-translational modification, targets them for recognition by the pVHL protein, leading to ubiquitination and proteasomal degradation [72].

Under hypoxic conditions, PHD enzymes become inactive due to lack of oxygen, allowing HIF-α to stabilize, dimerize with HIF-β (ARNT), and translocate to the nucleus. This complex binds to HREs in the EPO gene promoter, activating its transcription [70]. HIF-2α is the main isoform regulating EPO expression in renal peritubular cells. In contrast, HIF-1α plays broader roles in glycolytic metabolism, angiogenesis, and cellular adaptation to hypoxia. Animal models with HIF-2α knockout show abolished renal EPO expression, reinforcing its specificity [73].

Positive regulators: androgens, which increase EPO production, contributing to sex differences in hematocrit; IL-6 and other cytokines, which may enhance EPO expression under certain conditions; and HIF stabilizers (e.g., roxadustat, daprodustat), which inhibit PHDs and mimic hypoxia to induce EPO. Negative regulators: chronic inflammation—cytokines such as IL-1β and TNF-α suppress EPO transcription; and CKD—loss of EPO-producing cells leads to anemia [74].

The EPO gene is subject to epigenetic modifications including DNA methylation and histone modifications. Hypermethylation of the EPO promoter can repress its transcription. Histone acetylation and methylation patterns also influence chromatin accessibility and HIF binding [75].

## 6. Recombinant Erythropoietin in the Context of Gene Doping

Recent progress in genomics has facilitated the development of gene therapies aimed at correcting or replacing genes involved in essential physiological processes, thereby improving the production of biologically active compounds with limited natural activity. Such therapeutic applications include the stimulation of erythropoiesis and the biosynthesis of recombinant endogenous proteins, such as insulin [76].

However, unregulated manipulation of genetic material may induce significant metabolic disturbances that pose health risks. In this context, doping refers to the illicit use of pharmacological substances and methods—contrary to established regulations—with the intent to artificially enhance physical performance. The term “doping” first appeared in 1889 in a dictionary, describing a stimulant mixture containing opium, reportedly used to improve the performance of racehorses.

RHuEPO was originally developed to treat various forms of anemia but was later misappropriated for performance enhancement in sports. This misuse led to the development of novel EPOR agonists and mimetic compounds that mimic cytokine hormones while lacking a defined protein structure. To enhance detection and pharmacological properties, rHuEPO was biopharmaceutically modified by the addition of carbohydrate chains or polyethylene glycol, thereby refining testing methods used in standard anti-doping protocols [77].

The identification of doping agents presented further challenges due to the prolonged biological effects of anabolic steroids, which may exceed the detection window in biological samples.

Advances in the understanding of end-metabolite pharmacokinetics have improved the detection of steroid abuse. Simultaneously, doping practices evolved alongside research into novel compounds with performance-enhancing potential, including recombinant peptide hormones and related agents [76].

Testing methods recognized by the World Anti-Doping Agency (WADA), which incorporate additional biomarkers, have proven effective in enforcing anti-doping regulations in sports. These include the detection of transgenes, specific biochemical analytes, and characteristic serum steroid profiles. Ongoing research also addresses unintentional violations of anti-doping rules and the potential for misinterpretation of test results [78].

In gene therapy, targeting the genetic material of somatic cells is considered safer, as the modifications are limited to the individual and are not inherited, unlike alterations in germline cells, which result in heritable genetic changes. The transfer of genetic information can be accomplished through viral vectors—offering benefits such as efficient cell entry and replication—or through non-viral methods, such as electroporation, where an electric field increases cell membrane permeability and facilitates DNA uptake [76].

Techniques derived from genetic engineering and applied in gene doping have been shown to enhance both hematologic and anabolic parameters, leading to increases in muscle mass and physical strength. Specifically, overexpression of the EPO gene, which encodes a glycoprotein hormone, triggers physiological adaptations including elevated red blood cell count, enhanced oxygen-carrying capacity, and improved endurance performance.

Gene doping involves introducing the EPO gene into the body using viral vectors, leading to the overexpression of erythropoietin by encoding proteins that influence oxygen homeostasis. This artificial stimulation of erythropoiesis in the liver and kidneys enhances tissue oxygenation and increases hematocrit levels. However, such elevations in red blood cell count carry serious health risks, including the potential for stroke, myocardial infarction, thrombosis, and elevated peripheral vascular resistance [76].

Gene doping also presents significant concerns regarding adverse effects. These include unintended alterations in genetic material, such as mutations, or the potential for oncogenesis due to incorrect integration of the introduced gene into the host genome. Detecting gene doping remains a critical challenge. Current research efforts are focused on identifying the presence of viral vectors, analyzing gene expression profiles for doping-induced changes, conducting proteomic profiling, and distinguishing between naturally occurring proteins and those produced through recombinant or transgenic techniques.

The intake of protein products highlights the increase in resistance, physical strength, muscle mass, regulates the growth and/or regeneration of muscle tissue and encodes peptides that relieve pain [76]. RHuEPO was obtained by cloning the endogenous human EPO gene. The engineering process that was carried out included a post-translational modification, followed by a glycosylation process (binding of a carbohydrate molecule to the functional group of another molecule).

Clinical studies involving patients who were repeatedly administered rHuEPO recorded the development of antibodies that neutralize its contribution, a process that led to the appearance and development of PRCA [79]. PRCA induced by EPO should take into account when there is progressive anemia during EPO use, the combination of Roxadustat with immunotherapy being a more beneficial therapeutic alternative [80].

RHuEPO was marketed under various trade names, the synthesis of the product being related to the technology of the moment for obtaining the active substance. The treatment with rHuEPO depends on the health status of each patient and the specific therapeutic indications of the product used. The treatment is performed under the conditions of a medical staff specializing in the therapeutic approach to such situations. The therapeutic efficacy of rHuEPO could be determined by the ratio between hemoglobin level instability and the rate of reported adverse events.

The major aim of the treatment is to maintain Hb levels within the range documented in the patient’s medical history, eliminating subjective interpretations. Furthermore, the treatment with rHuEPO must be preceded by a complex and thorough evaluation of the user, the administration of various forms of iron supplements, to ensure an optimal response of the body to the prescribed treatment, knowing the possible side effects.

The beneficial effects of rHuEPO have expanded the scope of therapeutic hematopoietic and non-hematopoietic applications. The constant administration of rHuEPO has reduced the number of transfusions required in various surgical interventions and implicitly the risks related to donor issues. New ESAs have been developed, with an increased glycosylation potential, which has allowed the production of compounds with an extended half-life [79].

The therapeutic use of rHuEPO requires careful monitoring of the patient’s health prior to the decision to use it therapeutically and in accordance with approved clinical guidelines. Treatment with rHuEPO aims to achieve a recommended Hb range of 10–12 g/dL. Adjustment of the prescribed dose will 21 aim to achieve a Hb level between 10 g/dL–12 g/dL, with an initial dose of 50–150 units/kg, with a maintenance dose of 40 units/kg. If the Hb level exceeds 13 g/dL, treatment should be stopped until the Hb level falls below 13 g/dL, with a correction phase and a maintenance phase.

The treatment with rHuEPO for children usually requires higher doses than those for adults for a similar Hb response (doses decrease as the body weight and age of the children increase). This situation is determined by the plasma clearance rate (it relates to the speed of purification of a drug according to its concentration in biological fluids).

Clinical studies included in the rHuEPO use authorization programs have recommended monitoring of patients undergoing treatment in order to keep Hb levels within physiological limits, to avoid possible cardiovascular effects. In the event that Hb levels remain below physiological limits, patient monitoring will be based on individual case histories and the recommendations of the pharmaceutical manufacturers [81].

Advances in the pharmaceutical industry have allowed the administration of rHuEPO in a single dose over a week, using ESAs with an increased half-life, allowing the transition to a weekly dose. The use of a single weekly dose has in most cases shown a decrease in Hb levels, which has required the need to adjust the dose so as to reach the recommended physiological values, a situation that is not reported in clinically stable patients, who did not require an increase in the dose.

The adjustment of the correction dose to reach normal biological Hb values, in the case of hemodialysis-dependent patients, will be performed in stages, provided that one stage will take place over a minimum of 4 weeks [79]. Subcutaneous administration of rHuEPO by peritoneal dialysis has proven to be effective compared to hemodialysis, requiring a reduced amount of EPO. In order to ensure an improvement in the rigor of the treatment, the single dose version of rHuEPO is recommended.

Clinical administration of rHuEPO has highlighted physiological and metabolic changes related to EPO intake, the possible consequences of alternative treatment with an impact on the amplification of endogenous erythropoietin production.

The offers of the pharmaceutical industry highlight a dynamic in which the emergence of new biosimilars and new therapeutic modalities are related to discoveries in the field of molecular biology. These opportunities are aimed at differentiating products adapted to different life cycle options (aiming at improving therapeutic standards). The mechanism of the main rHuEPOs currently used is shown in Table 2.

The therapeutic use of rHuEPO requires monitoring possible side effects caused by its intake into the body, the medical particularities of each patient, and the recommendations of the pharmaceutical manufacturers for each product.

Multiple clinical studies that were the basis for obtaining the marketing authorization for rHuEPO under various trade names did not highlight the interactions of this compound with other drugs or other forms of interaction that would influence the metabolism of other drugs. The pharmacokinetic properties of rHuEPO are presented in Table 3.

RHuEPO therapy covers a very broad therapeutic spectrum, with specific features for each product, including the amplification of pharmacological effects in the event of overdose or very high plasma concentrations with side effects included in the manufacturer’s package insert. The safety data of rHuEPO are presented in Table 4. RHuEPO, marketed under various trade names, falls within the WHO system in the following pharmacotherapeutic group: other antianemic preparations, erythropoietin; ATC code (anatomical, therapeutic, chemical classification): B03XA01.

Furthermore, the mechanism of action of rHuEPO is involved in the phases of erythroid development, the development of erythroid precursors, binding to their receptors, and the activation of transduction channels that stimulate erythroid cell proliferation. Pharmacokinetic data provided by clinical studies have highlighted a series of specific common elements and particularities of each type of epoetin administered in relation to the specific characteristics of the user’s health condition. Toxicological studies have recorded preclinical safety data that allowed EMA or FDA approval. The ability of testing methods to detect rHuEpo is dependent on the performance of the assays, the ability to collect samples within a time frame that allows detection of the substances, and the doping methods used [89]. The results of the rHuEPO detection trials have been inconclusive, leaving room for further studies. These include determining the sensitivity and specificity of the sample with the isoelectric focusing (IEF) assay [59] or determining the sensitivity and specificity with the sarcosyl-PAGE assay [90].

## 7. Effect of EPO on Glucose Metabolism

While EPO is primarily known for its role in red blood cell production, recent studies have uncovered its metabolic effects beyond erythropoiesis.

EPO appears to exert beneficial effects on glucose metabolism by improving insulin sensitivity, enhancing glucose uptake, and modulating lipid metabolism.

These pleiotropic actions are of growing interest in the context of metabolic syndrome, obesity, and diabetes.

Recent studies revealed that EPO plays a role in glucose regulation independent of its erythropoietic function. EPO administration improves insulin sensitivity and glucose uptake in skeletal muscle and adipose tissue.

It enhances GLUT4 translocation, promotes mitochondrial biogenesis, and activates AMP-activated protein kinase (AMPK) and PGC-1α. These effects suggest a direct action of EPO on peripheral tissue glucose utilization and systemic metabolic health [68].

### 7.1. Mechanisms of EPO Action on Glucose Homeostasis

EPO acts on various peripheral tissues such as skeletal muscle, adipose tissue, and liver to regulate glucose metabolism.

The underlying mechanisms include activation of signaling pathways like PI3K/Akt, AMPK, and STAT5, which converge on key metabolic targets, including the stimulation of GLUT4 translocation to the plasma membrane; enhancement of mitochondrial biogenesis and oxidative phosphorylation; suppression of inflammatory cytokines in adipose tissue; and reduction in hepatic gluconeogenesis [22].

### 7.2. Tissue-Specific Effects of EPO

EPO enhances insulin-stimulated glucose uptake and utilization in skeletal muscle, partly via the PI3K/Akt and AMPK pathways. This contributes to improved systemic glucose tolerance.

In adipose tissue, EPO reduces macrophage infiltration and proinflammatory cytokine production, thereby improving insulin sensitivity and glucose utilization.

EPO has been shown to reduce gluconeogenic gene expression (e.g., PEPCK, G6Pase), contributing to lower hepatic glucose output. These effects may be mediated via activation of the AKT pathway.

Emerging evidence suggests EPO may exert cytoprotective effects on pancreatic β-cells, supporting their function and survival in the context of hyperglycemia or oxidative stress [91].

### 7.3. Metabolic Effects of EPO in Mouse Models

In mouse models of obesity and insulin resistance, EPO administration led to improved glucose tolerance and insulin sensitivity. These effects were independent of hematocrit changes, suggesting a direct metabolic role. In vitro studies in myotubes and adipocytes have confirmed EPO’s ability to stimulate glucose uptake and enhance mitochondrial respiration [92].

### 7.4. Clinical Implications

Although (rHuEPO) is not approved for treating metabolic diseases, its metabolic effects are of interest for potential therapeutic strategies against type 2 diabetes and metabolic syndrome [93,94].

HIF stabilizers, which also increase endogenous EPO levels, may have additional metabolic benefits that warrant further investigation [68].

## 8. EPO as a Regulator of Metabolic Energy Pathways

While EPO is classically recognized for stimulating erythropoiesis, recent studies have highlighted its broader role as a regulator of systemic energy metabolism, including the modulation of the overall metabolic balance [22,68]. Beyond its hematopoietic function, EPO acts on several metabolically active tissues, including adipose tissue, skeletal muscle, liver, and even the central nervous system, to coordinate glucose and lipid homeostasis, mitochondrial function, and overall energy expenditure [22,55].

Beyond its role in red cell mass regulation, EPO has been implicated in systemic energy balance. EPO stimulates fatty acid oxidation and suppresses lipid accumulation.

It promotes browning of white adipose tissue and enhances mitochondrial oxidative capacity. These effects are mediated through transcriptional regulators such as PGC-1α, SIRT1, and NRF1.

The hormone is also thought to influence hypothalamic neurons, potentially affecting appetite and energy expenditure [95].

### 8.1. Molecular Mechanisms of EPO in Energy Regulation

EPO mediates its metabolic effects through EPORs, which activates downstream signaling pathways such as: Janus kinase 2 (JAK2)/Signal Transducer and Activator of Transcription 5 (STAT5); Phosphatidylinositol 3-kinase (PI3K)/Akt pathway; AMP-activated protein kinase (AMPK); and Mammalian target of rapamycin (mTOR) [96].

These signaling cascades ultimately influence mitochondrial biogenesis, oxidative phosphorylation, and substrate metabolism. EPO has been shown to increase mitochondrial content and improve efficiency in several tissues, thereby enhancing aerobic capacity and energy utilization [95].

### 8.2. Tissue-Specific Effects of EPO on Energy Metabolism

EPO promotes mitochondrial biogenesis, oxidative phosphorylation, and fatty acid oxidation in muscle cells. These effects contribute to increased endurance and reduced fat accumulation. EPO also upregulates the expression of PGC-1α, a master regulator of mitochondrial biogenesis [96].

In white adipose tissue, EPO reduces inflammation, enhances mitochondrial function, and stimulates browning of fat. This transition from white to beige adipocytes increases thermogenic activity and energy expenditure [68].

EPO modulates hepatic lipid metabolism by reducing triglyceride accumulation and improving insulin sensitivity. These actions help ameliorate hepatic steatosis in models of metabolic syndrome [55].

In the hypothalamus, EPO may regulate appetite and systemic energy expenditure via neuronal EPOR signaling. Animal studies suggest EPO influences leptin sensitivity and neuroinflammation [68].

### 8.3. Experimental and Clinical Evidence

Multiple animal studies have demonstrated that EPO administration improves metabolic parameters in obese and insulin-resistant mice [68]. These effects include increased oxygen consumption, enhanced mitochondrial density, reduced body weight, and improved glucose and lipid profiles.

Although data in humans are limited, some studies in patients with chronic kidney disease (CKD) receiving recombinant human EPO have reported improved metabolic markers, suggesting potential translational relevance [96].

### 8.4. Therapeutic Implications

Understanding EPO’s role in energy regulation opens the door to potential new therapeutic strategies for metabolic diseases. Pharmacological agents that enhance endogenous EPO production (e.g., HIF-PHD inhibitors) may offer dual benefits in anemia and energy metabolism disorders, though risks like erythrocytosis must be carefully managed [55]. Recent studies have also suggested that HIF-PHs are effective in improving anemia in patients with heart failure or patients suffering from CKD. A systematic meta-analysis of four studies (including 98 patients) suggested that treatment with HIF-PH inhibitors increased Hb levels by 0.7–0.8 g/dL over one to three months (without significantly affecting the renal function or heart failure biomarkers such as NT-proBNP-B-type pronatriuretic peptide). No sever adverse effects were detected (indicating a safe short-term safety profile) [97]. Moreover, HIF-PH inhibitors have also marked a major advance in the treatment of anemia of chronic disease (ACD). HIF-PH inhibitors are efficient and safe for treating CKD and tumor-associated anemia and are being explored for broader indications such as post-renal transplant anemia, aplastic anemia, multiple myeloma, and anemia linked to autoimmune diseases or infections. Preclinical studies indicate their potential benefit in treating ischemic conditions such as kidney injury or stroke. Nonetheless, important questions remain regarding their long-term safety (in tumor settings), thrombotic and cardiovascular risks, and their efficacy in patients with micro-inflammation who are responsive to conventional ESAs. Further studies are needed in order to clarify their broader applicability and long-term outcomes [98].

## 9. Pharmacological Insights into Blood Doping and Its Physiological Implications

Blood doping is defined as an attempt to enhance performance by various methods that are not subjective to regulations. The attempt consists of using medical procedures such as blood transfusions and pharmaceutical products such as rHuEPO to enhance the capacity and endurance of athletes. The repercussions caused are the following: stimulation of red blood cell production and additional supply of oxygen to the muscle level by intervening on HIF; use of autologous blood transfusion, by collecting, processing and reinfusing one’s own blood, eliminating possible side effects; and use of artificial oxygen transporters at the tissue level, improving the oxygen supply to the muscular system. The most commonly used procedures in blood doping since 1989 remain the use of blood transfusions and the administration of rHuEPO [99]. The use of rHuEPO has been shown to enhance various cardiopulmonary-related factors that can ultimately boost the endurance performance. RHuEPO is able to affect the physiological, hematological, and performance metrics in well-trained athletes participating in endurance activities. Regardless of what dose is given, rHuEPO can improve hematological parameters such as Hb and hematocrit. As a result, rHuEPO has the ability to enhance oxygen transport and to boost the erythrocyte mass, providing a physiological benefit to athletes who misuse it [100]. Analysis of blood rheology, of the viscosity of the blood flow received at the tissue level or of the physiology of red cells, under conditions of prolonged effort, has highlighted aspects (e.g., red blood cell senescence, etc.) whose pharmacological interpretation has been found in the use of new doping procedures. Solving these challenges has led to the need for new analytical procedures and technologies for doping detection [99]. Efforts to combat blood doping include improving the analytical sensitivity of test methods and interpreting their profiles, correctly interpreting data in terms of biochemical and energetic changes, detecting differences between endogenous and synthetic compounds and identifying complementary matrices that include parameters with doping potential (e.g., dried blood spots) in the absence of EPO signals in urine [101]. WADA has recently adopted dried blood spots (DBSs) as a method for doping control [102]. The physiological aspects related to the development of blood doping are relevant in terms of the role played by the participants in this process. Hb is able to transport oxygen from the lungs to the tissues and takes carbon dioxide from the tissues, transporting it to the lungs, increasing thus the blood’s ability to transport oxygen. The dissociation of oxygen in tissues is ensured by the signal at low oxygen levels (enhancing the erythropoiesis process). HIFs induce the production of EPO or other genes under conditions of low oxygen levels (by controlling the rate of transcription of genetic information). Furthermore, EPORs are found predominantly expressed on the hematopoietic stem of cells in the bone marrow. In this whole process reticulocytes are released, becoming mature erythrocytes.

Blood transfusion is a medical procedure, an intravenous transfer, that consists of administering by injection to a person blood product from a donor (allogeneic transfusion) or from the same person (autotransfusion). The use of transfusion in the medical field has also shown interest to those interested in blood doping, with autotransfusion becoming a very frequently reported method of blood doping, given that it is much more difficult to detect. Autotransfusion, performed under inadequate conditions, poses risks, as it can cause volume overload or a reaction caused by antigen mismatch, causing the appearance of an immune response aimed at eliminating or neutralizing it [103].

**Figure 3:**
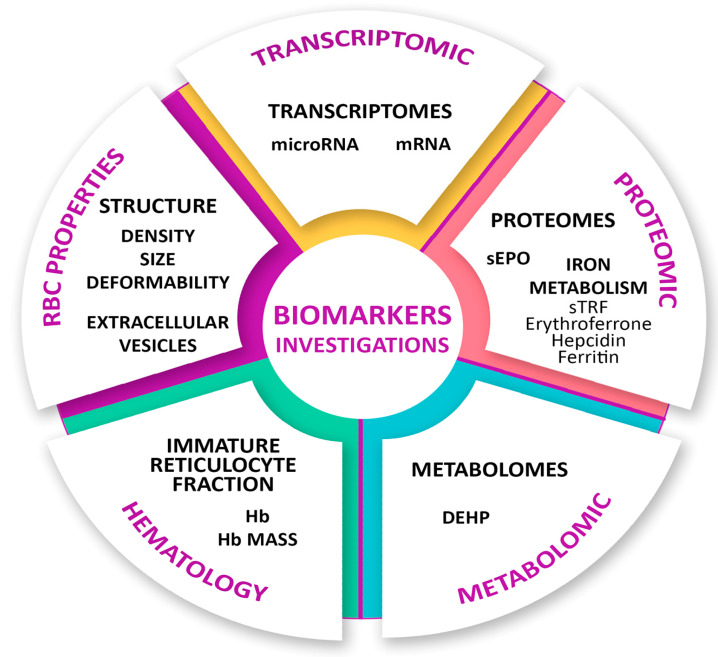
Biomarkers used in the detection of performance-enhancing substances [105]. Multi-level biomarkers used for detecting performance-enhancing substances, including: transcriptomic markers, proteomic markers, metabolomic markers, hematological parameters and red blood cell properties. These biomarkers provide an integrated view of physiological responses to doping.

Blood doping attempts with homologous erythrocytes are easy to detect due to antigenic differences between the user and the donor. The cells are subjected to flow cytometric analysis. Several biomarkers are currently being investigated to highlight the use of autotransfusion as a doping method: analysis of circulating microRNA, changes in hepcidin, changes in biomarkers associated with the production of transcription during the biological process of message transcription, analysis of red microparticles in the blood. The presence in the urine collected from users of components existing in the plastic bags (in which the blood is stored) or of components existing in the plastic tubes (used in blood transfusion as a doping method) can be detected after the transfusion, indicating the use of blood doping as a fraudulent alternative to obtain performance in sports [104]. The main biomarkers researched in sports doping are showcased in Figure 3 [105].

Recent evidence highlights the potential role for the microNRA miR-223 in mediating the effects of uremic toxins on anemia in CKD patients. Uremic toxins such as indoxyl sulfate (IS) are building up in the bloodstream and are commonly associated with anemia in CKD. IS can affect the production of red blood cells in lab and animal models, thus impairing the effectiveness of EPO therapy. Both IS and microRNA miR-223 are linked to anemia in CKD. Many tests were conducted on human red blood cells precursors (UT7/EPO cells) and indicated that IS can cause an increase in miR-223. The same results were also confirmed in human CD34+ cells. Higher levels of miR-223 in the bloodstream are linked to blood vessel damage. As a result, miR-223 can contribute to anemia in CKD patients, possibly interfering with red blood cell production. Understanding this mechanism could lead to new treatments for anemia in CKD patients. miR-223 can serve as a biomarker for the early detection and monitoring of anemia-related cardiovascular disease during CKD progression. Understanding this pathway could help explain why some patients respond poorly to EPO treatments and could suggest new therapeutic strategies for, e.g., modulating miR-223 to improve EPO efficacy or reduce side effects [106].

A variety of biomarkers are currently under investigation for their utility in detecting doping practices in sports. Among these, transcriptomic markers are particularly valuable for identifying early molecular responses to prohibited substances, as they capture gene expression changes that precede phenotypic alterations. Proteomic markers, on the other hand, provide insight into the modulation of specific protein levels associated with enhanced erythropoiesis or altered metabolism. Another critical category includes iron metabolism markers, which are indicative of disruptions in iron absorption, transport, and utilization—frequent targets of erythropoiesis-stimulating agents such as recombinant erythropoietin.

Furthermore, metabolomic profiling enables the detection of shifts in small-molecule concentrations within biological fluids, offering a sensitive window into systemic metabolic changes induced by doping agents.

Traditional hematological markers, such as Hb concentration and reticulocyte count, remain central to anti-doping strategies, particularly through integration into longitudinal monitoring frameworks like the Athlete Biological Passport. Lastly, alterations in red blood cell properties, including their mechanical flexibility and density, are increasingly being studied as indirect indicators of blood manipulation or pharmacologically enhanced erythropoiesis.

## 10. The Athlete’s Biological Passport

The Athlete Biological Passport (ABP) is a concept introduced by WADA in 2009, aimed at identifying biological variables that require special attention, showcasing anti-doping investigations and testing and monitoring possible violations of doping regulations.

The ABP is composed of a hematological and steroid module. Blood doping can alter the hematological parameters by increasing the reticulocyte, hematocrit counts, and EPO levels due to the amplification of red cell mass [107]. The data provided by the ABP are incomplete but can be corroborated by data provided by classical testing (e.g., decrease in test sensitivity beyond a certain testing window, etc.) [108]. Blood doping practices, beyond awareness campaigns of the consequences of its use or the performance of current detection methods, remain relevant [109].

The analytical methods used to detect the use of autotransfusion in blood doping aim to detect the presence of a new population of erythrocytes in the body of the person who has undergone such practices [110].

Autologous transfusion involves the collection, storage, and subsequent reinfusion of one’s own blood, aiming to improve the number of red blood cells in the bloodstream [111]. The results of a pilot study by Bizjak et al. [112] documented aspects related to the cryopreservation of blood samples, the course of autologous transfusion, cell deformability and viscosity, and the values of analytes associated with nitric oxide in cells.

The values of metabolic parameters and electrolyte concentrations remained constant, which was attributed to the low volume of reinfused blood [112]. Other results of specialized studies conducted by Miller et al. considered atypical biomarker values, thus supporting revisions to the ABP’s profile [113]. Garvican-Lewis et al. proposed a model to monitor the variation in plasma levels and eligible values of blood parameters, under conditions of induced hemodilution, brought an improvement in the interpretation of data [114]. Another relevant study conducted by Voss et al. monitored the values of biological biomarkers recording atypical changes in the hematological module (e.g., reticulocyte percentage, hemoglobin concentration, OFF score, etc.) [115]. The periodic updating of the ABP aims to establish clear reference criteria regarding the strategy and calendar of sample collection [116].

Establishing an effective and credible testing system is fundamental to the integrity and efficacy of anti-doping efforts. Nevertheless, the relatively low rate of detection compared to the estimated actual prevalence of doping in sports has led to growing skepticism and criticism regarding the efficiency of current testing frameworks. To be effective, anti-doping testing strategies must distinguish between preventive testing and targeted testing aimed at detecting the use of prohibited substances or methods.

When the primary goal is detection, testing protocols should be driven by risk assessment and intelligence gathering, prioritizing the strategic quality of testing plans rather than the sheer volume of samples collected. In this context, the detection rate is a key performance indicator for evaluating the success of testing programs.

Importantly, the number of individual athletes tested—rather than the total number of samples—provides a more accurate reflection of a testing program’s ability to identify doping behavior [117].

The implementation of comprehensive anti-doping regulations serves to uphold fairness, transparency, and the protection of athlete health. In this regard, healthcare professionals, particularly pharmacists, can play a pivotal role in doping prevention. The strong rapport between athletes and healthcare providers facilitates the dissemination of accurate information, development of life skills, and promotion of informed decision-making, ultimately reducing both intentional and unintentional anti-doping rule violations [118]. Patterns of pharmaceutical use among athletes further underscore the need for vigilance [119]. The development and application of specific biomarkers hold promise for enhancing the detection of synthetic or modified EPO and structurally related doping agents in urinary samples, representing a crucial advancement in anti-doping science [120].

## 11. Regulatory Role of Erythropoietin in Iron Metabolism and Homeostasis

Iron distribution and accumulation within the human body play a fundamental role in the regulation of erythropoiesis. Total body iron content typically ranges from 3 to 5 g, partitioned among ferritin stores—primarily hepatic (approximately 20%), hemoglobin within circulating erythrocytes (around 70%), and enzymatic or other protein-bound forms (about 10%) [121]. In healthy individuals, iron homeostasis is maintained through the absorption of 1–2 mg of dietary iron per day, compensating for physiological losses such as epithelial desquamation and intestinal shedding; in women, menstrual bleeding represents an additional loss [121].

Ferritin concentrations serve as key indicators of iron storage status. Low ferritin levels reflect depleted iron reserves, often culminating in iron-deficiency anemia, whereas elevated levels are associated with iron overload conditions, such as liver and cardiac diseases, chronic inflammation, or infection [122]. Since the body lacks robust mechanisms for iron excretion, its homeostasis is largely regulated at the level of intestinal absorption. Dietary iron is primarily absorbed in the duodenum and jejunum and transported by transferrin, with approximately 80% of absorbed iron directed to the bone marrow to support erythropoiesis.

EPO significantly accelerates erythropoiesis. However, studies have demonstrated that exogenous administration of EPO can disrupt iron homeostasis. Notably, plasma ferritin levels may not accurately reflect total iron stores in the context of recombinant EPO administration [123]. The supraphysiologic stimulation of erythropoiesis through injectable EPO may present with paradoxical findings, including low hemoglobin concentrations, apparent iron deficiency, or impaired mobilization of stored iron [124]. EPO modulates systemic iron metabolism by downregulating hepcidin—a central regulator of iron absorption and mobilization—thereby enhancing intestinal iron uptake, reducing ferritin levels, and promoting hemoglobin synthesis [123].

Differentiating the effects of various ESAs on iron utilization underscores the need for advanced analytical frameworks to monitor treatment responses. For instance, correlations between iron supplementation routes (oral vs. intravenous) and plasma hepcidin modulation offer valuable insights into ESA efficacy and iron handling [123]. Evaluating the impact of EPO requires a comprehensive assessment that incorporates safety and efficacy profiles, especially in relation to the sensitivity and cut-off thresholds of ferritin assays, which remain insufficiently defined [124].

Given the complexity of iron regulation under ESA therapy, ongoing refinement of biomarker panels is essential. Current iron indices provide incomplete data for some patient populations, thus underscoring the need for additional biomarkers to improve diagnostic precision [125,126].

While EPO and related therapies reduce the incidence of adverse hematologic events, their effects on ferritin and serum iron levels necessitate a precise understanding of iron homeostasis and its therapeutic manipulation.

Iron plays a pivotal role in cellular functions, and imbalances in its metabolism are linked to pathological states. Iron overload is associated with hereditary hemochromatosis and thalassemia, whereas deficiency results in various forms of anemia. Excessive iron contributes to oxidative stress via increased production of reactive oxygen species, ultimately causing tissue damage and increasing the risk of neurodegenerative, hepatic, pancreatic, and cardiovascular disorders [127].

In the case of inadequate iron intake, Hb synthesis is impaired, leading to diminished oxygen transport capacity. Restoring iron stores through dietary supplementation supports erythropoiesis and maintains systemic oxygen delivery [124].

Alcohol consumption has been shown to influence iron metabolism. In a study involving the intake of 300 mL of red wine daily for three weeks (preceded by a two-week abstention), reductions in serum hepcidin were observed in both healthy individuals and patients with type 2 diabetes, accompanied by increased EPO levels. Erythroferrone levels rose significantly in the diabetic cohort, indicating activation of the EPO–erythroferrone–hepcidin axis.

These molecular changes correlated with an increase in red cell distribution width in both groups and higher reticulocyte counts in diabetic participants. This suggests that moderate red wine consumption may positively influence iron regulation through this hormonal pathway [128].

These findings support the hypothesis that moderate red wine intake may confer cardiometabolic benefits, particularly in individuals with type 2 diabetes, as suggested by numerous epidemiological and interventional studies in humans. Such effects may contribute to the prevention or amelioration of diet-related non-communicable diseases, including cardiovascular disorders and diabetes mellitus type 2 [128].

## 12. Cutting-Edge Perspectives on Erythropoietin Mechanisms and Use

EPO ranks among the costliest elements used in the treatment of anemia. To overcome this inconvenience researchers were able to create synthetic EPO receptors by employing design-build-test cycles alongside genome editing. The new synthetic receptors are able to enable the erythropoiesis process. Thus, the production of red blood cells can be more affordable [129]. EPO has been discovered to have an immunosuppressive function in liver cancer (hepatocellular carcinoma, HCC).

As a result, EPO produced by tumors can stimulate the EPO/EPOR signaling pathway in macrophages found in a tumor microenvironment (TME). Because of that, the immune response is diminished by decreasing the T cell infiltration and functionality.

Consequently, the EPO/EPOR pathway represents a significant barrier to effective antitumor immunity and is under exploration as a possible therapeutic target to boost immune responses and enhance treatment results in HCC.

**Figure 4:**
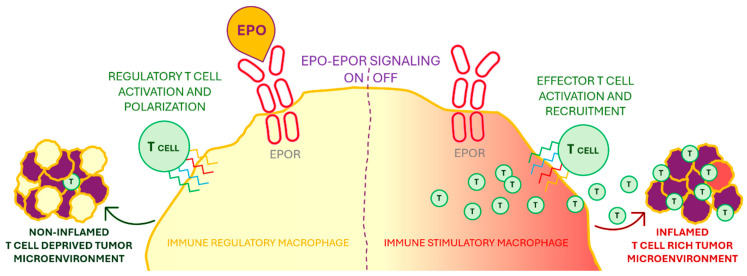
Macrophage signaling pathways that promote a non-inflamed tumor immune phenotype [130]. Macrophages shape the tumor immune enviroment by activating regulatory T cells and suppresing effector T cells. EPO–EPOR signaling modulates this process by determining whether the tumor remains immune-suppressed or becomes inflammed and T cell-rich (supporting anti-tumor responses).

The EPO/EPOR signaling in macrophages that is able to create a noninflamed tumor immunotype is presented in Figure 4 [130].

EPO signaling plays a critical role in shaping the tumor immune microenvironment (TME), particularly through its effects on macrophage polarization. Tumor-derived EPO can bind to EPORs expressed on macrophages, inducing an immunosuppressive phenotype. This shift promotes the expansion of regulatory T cells (Tregs), suppresses proinflammatory responses, and contributes to the establishment of an immunologically “cold” TME, which impairs effective anti-tumor immunity. Conversely, inhibition of the EPO–EPOR axis reprograms macrophages toward a proinflammatory state, fostering a “hot” TME that supports T-cell activation and anti-tumoral responses.

Clinically, ESAs are approved for the management of chemotherapy-induced anemia in patients with nonhematologic malignancies, aiming to reduce the need for red blood cell (RBC) transfusions. However, ESA use is associated with an increased risk of thromboembolic complications and elevated mortality. To mitigate these risks, guidelines recommend initiating treatment at Hb levels below 10 g/dL and avoiding Hb levels above 12 g/dL during therapy [131].

Beyond oncology, EPO is extensively utilized in the treatment of anemia associated with CKD. It contributes to metabolic homeostasis by promoting lipolysis and suppressing lipogenic gene expression [68]. Updated therapeutic guidelines have removed target Hb thresholds for CKD-related anemia, instead advocating for the lowest effective ESA dose to avoid transfusions. Biosimilar ESAs have demonstrated equivalent safety and efficacy to originator products across non-U.S. markets [132].

Daprodustat, a novel HIF-PHD inhibitor recently approved by the FDA, exhibits non-inferiority to rHuEPO for anemia treatment in hemodialysis patients [133]. Similarly, darbepoetin and EPO have shown comparable safety and efficacy profiles in CKD-related anemia management [134].

Hemodialysis patients with advanced CKD are particularly vulnerable to SARS-CoV-2, where inflammation-induced EPO resistance complicates anemia treatment. Temporary EPO resistance during COVID-19 infection necessitates personalized therapeutic strategies, including the use of HIF stabilizers [135]. In this context, EPO’s pleiotropic benefits may alleviate COVID-19-related complications by supporting respiration, modulating neuroinflammation, and promoting neuroregeneration [136,137].

Beyond hematopoiesis, EPO exhibits tissue-protective properties through interaction with the tissue-protective receptor (TPR), suppressing proinflammatory cytokines and inhibiting apoptosis. It enhances wound healing and rebalances T helper cell responses [138]. In endocrine pathologies such as primary hyperparathyroidism (PHPT), EPO resistance is mediated by elevated fibroblast growth factor 23 (FGF23), a hormone implicated in phosphate homeostasis, inflammation, and erythropoiesis [139,140].

Genetic variations in the EPO and EPOR loci have been associated with erythrocytosis, defined by elevated RBC mass, hematocrit, and hemoglobin levels. Primary erythrocytosis, as seen in polycythemia vera, presents with suppressed EPO due to intrinsic hematopoietic stem cell abnormalities, while secondary erythrocytosis is marked by elevated or inappropriately normal EPO in response to hypoxia or ectopic production [141,142].

Elevated EPO levels have also been observed in sepsis, where its anti-inflammatory and cytoprotective roles help attenuate immune dysregulation, oxidative stress, and tissue injury. Non-survivors of sepsis often exhibit higher endogenous EPO concentrations, correlating with tissue hypoperfusion [143].

Neuroprotective applications of EPO have been investigated in neonatal hypoxic–ischemic encephalopathy (HIE). While some studies suggest benefits when used alone or in combination with therapeutic hypothermia (TH), others report limited impact on mortality or neurodevelopmental outcomes, with some studies even indicating increased adverse events [144,145,146,147]. Nevertheless, in resource-limited settings lacking access to TH, EPO has demonstrated promising neuroprotective and behavioral benefits [148,149]. Preclinical and early clinical studies suggest that combining EPO with TH and neuroprotectants such as melatonin may enhance outcomes following perinatal brain injury [150,151].

EPOR expression in both normal and malignant tissues implicates it in mitochondrial regulation and tumor progression. In cancer cells, EPOR signaling modulates mitochondrial biogenesis via pAKT and inducible nitric oxide synthase (iNOS), highlighting its significance in tumor bioenergetics [152].

During pregnancy, EPO is utilized to manage iron-deficiency anemia, often in conjunction with iron supplementation to optimize hematologic outcomes [153]. Similarly, in autoimmune hemolytic anemia (AIHA) with inadequate bone marrow compensation, rHuEPO alongside immunosuppressive therapy can restore erythropoiesis and reduce transfusion dependence [154].

Despite its central role in oxygen homeostasis and erythropoiesis, the molecular underpinnings of renal EPO production remain incompletely characterized, limiting therapeutic advancements for anemia [155].

Glucocorticoids (GCs) also modulate erythropoiesis by enhancing EPO sensitivity in progenitor cells and promoting proliferation under stress. Their influence is evident in disorders such as Diamond-Blackfan anemia and myelodysplastic syndromes. Genetic and epigenetic determinants of GC responsiveness remain under investigation. GC deficiency in Addison’s disease results in anemia, while excess in Cushing’s syndrome may cause erythrocytosis [156]. In states of injury or infection, stress erythropoiesis temporarily compensates for impaired steady-state erythropoiesis. Although EPO does not initiate this pathway, it supports and amplifies its response [62].

Finally, EPO expression is influenced not only by hypoxia through the oxygen- sensing pathway but also by hormonal regulation via the renin–angiotensin–aldosterone system (RAAS), with angiotensin II upregulating EPO mRNA and protein in renal tubules [157,158].

## 13. Conclusions

The development of erythropoiesis, within normal parameters, depends on the supply of a protein hormone, EPO [159].

The production of EPO in the body is induced under conditions of deficient oxygen supply to the tissues (caused by biological dysfunctions that affect the erythropoiesis process). Research on substances with doping potential requires a permanent effort that involves: reporting on current innovations and technology, monitoring aspects related to analytical sensitivity, exhaustiveness of target analytes, differentiating natural/endogenous substances from structurally identical ones or differentiating synthetic derived compounds. Routine anti-doping controls consider testing biological samples. The data found in the matrix of the collected samples allow monitoring of organic and inorganic analytes with various molecular masses. Diversification of anti-doping testing instruments and methods aims to analyze and evaluate new parameters found in an alternative matrix. The continuous optimization of analytical approaches, using innovative processes and technologies, aims to understand the final and intermediate products of metabolic transformations. The optimal ranges for detecting compounds with doping potential are determined using liquid chromatography-tandem mass spectrometry-based assays. These aspects highlight the complexity and scale of the challenges in the effort to detect these substances (e.g., steroid hormones, erythropoiesis-stimulating agents, target analytes of gene doping, anabolic agents, peptide hormones, stimulants, etc.). The concerns in the field have targeted innovative technologies for detecting compounds with doping potential (e.g., remote testing, reproduction of metabolic systems, profiling of serum steroids or exploring gene editing methods, etc.). The intake of ergogenic supplements initially had a therapeutic role; later, they were used in doping practices. The effects induced by erythropoietin and other erythropoiesis-stimulating agents on the problem of iron assimilation and availability and the erythropoiesis process shape the way to new analytical approaches. The management of therapeutic aspects related to iron deficiency in the body is reported to the use of rHuEPO, other ESAs or iron-based supplements, achieving stimulation of the erythropoiesis process and iron availability in the body, reductions in ferritin levels (a form of iron storage in the body), and increases in serum iron levels in the body [160].

Personalized medicine is reshaping the landscape of EPO therapy. A deep understanding of EPO’s pleiotropic mechanisms, genetic determinants of response, and emerging pharmacological tools will enable more effective, safer, and patient-centric treatments. Future research should aim to refine predictive models and expand therapeutic applications of EPO in both hematologic and non-hematologic domains.

## Figures and Tables

**Table 1:** Methodological Steps in ESA Development [10].

	Trade Name	License Holder	Year of Approval	Manufacturing Process
**First Generation**				
Epoietin alfa	Epogen^®^—US	Amgen	1989	Recombinant DNA technology (in CHO cells) ***
	Eprex^®^—Europe	Orto Biotech	1988	
	Procrit^®^—US	Amgen	1989	
**Second Generation**				
Epoietin beta	NeoRecormon^®^—Europe	Roche	1997	Recombinant DNA technology (in CHO cells) ***
	Recormon^®^—US	Roche	1997	
Darbepoetin alfa	Aranesp^®^—US, Europe	Amgen	2001	
**Third Generation**				
Epoietin delta	Dynepo® *—Europe	Transkaryotic Therapies/Shire	2007	Activated gene technology (in HT cells) **
Methoxy polyethylene glycol epoetin beta	Mircera®—US, Europe	Roche Registration GmbH	2007	Recombinant DNA technology (in CHO cells) ***
Epoetin alfa (biosimilar)	Binocrit^®^—Europe	Sandoz	2007	
	Abseamed^®^—Europe	Medice		
	Epoietin alfa-hexal^®^—Europe	Hexal AG		
	Retacrit^®^—US	Pfizer	2018	
Epoetin zeta (biosimilar)	Retacrit^®^—US, Europe	Hospiro	2007	
	Silapo^®^—Europe	Stada		
Epoetin theta	Eporatio^®^—Europe	Teva	2009	
	Biopoin^® *^—Europe	Ratio Pharm		

* Currently not approved by EMA. ** Human tumor. *** Chinese hamster ovary derived cell culture medium.

**Table 2:** Mechanism of action of currently licensed rHuEPOs [81].

Drug/Trade Name	Key Pharmacological Distinction
Epoetin beta (NeoRecormon^®^)	Composition identical to human urinary EPO from anemic patients; stimulates erythropoiesis without affecting leukopoiesis.
Darbepoetin alfa (Aranesp^®^)	Expression similar to endogenous EPO; longer half-life, reducing the number of required doses.
Epoetin beta (Mircera^®^)	PEGylated (methoxy-polyethylene attachment); structure identical to endogenous EPO but binds receptors differently, giving it a longer duration of action.
Epoetin alfa (Epoetin alfa XEXAL^®^)	165 amino acid sequence identical to endogenous epoetin; indistinguishable from natural EPO.
Epoetin alfa (Binocrit^®^)	Biosimilar to endogenous EPO; stimulates red blood cell production, with reported benefits but some potential risks.
Epoetin zeta (Retacrit^®^)	Biosimilar with 165 amino acid sequence identical to urinary EPO; acts as a growth factor stimulating erythropoiesis.
Epoetin theta (Eporatio^®^)	Produced via recombinant DNA technology; expressed similarly to endogenous EPO; stimulates red blood cell production.

**Table 3:** The main pharmacokinetic properties of currently licensed rHuEPO [82,83,84,85,86,87,88].

Drug/Trade Name	Pharmacokinetic/Distribution Characteristics	Hematological Parameter Variability	Other Notes
Epoetin beta (NeoRecormon^®^)	Peak serum concentration after 12–28 h	Varies with subject health status/diagnosis and route of administration	Amino acid and hydrocarbon composition identical to urinary EPO from anemic patients
Darbepoetin alfa (Aranesp^®^)	Uniform PK in adults & children/adolescents	Hematological parameters stable, independent of dose number & administration route	Longer half-life reduces injection frequency
Epoetin beta (Mircera^®^)	Modified with methoxy-polyethylene glycol (PEG); long-acting	Varies with health status/diagnosis and administration route; not influenced by hemodialysis	Binds differently to EPOR → prolonged action
Epoetin alfa (Epoetin alfa XEXAL^®^)	Slightly elevated distribution volume vs. plasma space	Hematological parameters vary with dose number, independent of route	Uniform PK in adults & children/adolescents
Epoetin alfa (Binocrit^®^)	Slightly elevated distribution volume vs. plasma space	Hematological parameters vary with dose number, independent of route	Uniform PK in adults & children/adolescents; biosimilar
Epoetin zeta (Retacrit^®^)	Distribution volume varies with diagnosis, dosage frequency, and route	Hematological parameters vary with dose number	Serum concentration increase (linear/nonlinear) influenced by diagnosis, age, and treatment
Epoetin theta (Eporatio^®^)	Peak serum concentration after 10–14 h	Hematological factors vary with gender, age, and administration method	Recombinant DNA-derived

**Table 4:** The main preclinical safety data following rHuEPO administration [82,83,84,85,86,87,88].

Drug/Trade Name	Preclinical Safety Data
Epoetin beta (NeoRecormon^®^)	Repeated doses did not reveal proliferative risks (e.g., no genotoxicity, carcinogenicity).
Darbepoetin alfa (Aranesp^®^)	Repeated doses showed enhanced pharmacological effects, ↑ blood viscosity, ↓ tissue permeability. No genotoxic, tumorigenic, or carcinogenic effects. No fertility impairment reported.
Epoetin beta (Mircera^®^)	No genotoxic, tumorigenic, or carcinogenic effects. No fertility impairment reported.
Epoetin alfa (Epoetin alfa XEXAL^®^)	Preclinical safety profile similar to epoetin zeta.
Epoetin alfa (Binocrit^®^)	Preclinical safety profile similar to epoetin alfa XEXAL^®^.
Epoetin zeta (Retacrit^®^)	Stimulates erythropoiesis without affecting leukopoiesis. Does not induce chromosomal aberrations or genetic mutations. Conflicting data on possible association with bone marrow fibrosis in secondary hyperparathyroidism.
Epoetin theta (Eporatio^®^)	No genotoxic, tumorigenic, or carcinogenic effects.

Notes: ↑ blood viscosity, high blood viscosity; ↓ tissue permeability, low tissue permeability.
